# Advanced esophagogastric junction mixed neuroendocrine–non-neuroendocrine neoplasm with long-term recurrence-free survival

**DOI:** 10.1186/s40792-024-02011-8

**Published:** 2024-09-11

**Authors:** Shunsuke Takenaka, Toshikatsu Tsuji, Kenta Doden, Saki Hayashi, Mari Shimada, Hiroto Saito, Daisuke Yamamoto, Koichi Okamoto, Hiroko Ikeda, Hideki Moriyama, Jun Kinoshita, Yasunori Sato, Itasu Ninomiya, Noriyuki Inaki

**Affiliations:** 1 Department of Gastrointestinal Surgery, Kanazawa University Hospital, 13-1 Takara-Machi, Kanazawa, Ishikawa 920-8641 Japan; 2 Department of General and Digestive Surgery, Kanazawa Medical University Hospital, 1-1 Daigaku, Uchinada, Kahoku, Ishikawa 920-0293 Japan; 3 Department of Pathology, Kanazawa University Hospital, 13-1 Takara-Machi, Kanazawa, Ishikawa 920-8641 Japan; 4 Department of Human Pathology, Kanazawa University Graduate School of Medicine, 13-1 Takara-Machi, Kanazawa, Ishikawa 920-8641 Japan; 5 Department of Surgery, Fukui Prefectural Hospital, 2‐8‐1 Yotsui, Fukui, Fukui 910‐8526 Japan

**Keywords:** Mixed neuroendocrine–non-neuroendocrine neoplasm, Esophagogastric MiNEN, Recurrence-free survival

## Abstract

**Background:**

Mixed neuroendocrine–non-neuroendocrine neoplasm (MiNEN) is a rare malignant gastrointestinal tumor. The prognosis of patients with MiNEN is poor because of the high frequency of recurrence and metastases. We report a case of esophagogastric junction MiNEN (EGJ-MiNEN) with a long-term recurrence-free survival of 5.5 years.

**Case presentation:**

A 58-year-old male patient underwent thoracoscopic esophagectomy for esophagogastric junction adenocarcinoma. The patient’s postoperative course was uneventful. R0 resection was achieved, and the pathological diagnosis of the surgical specimen was pT3N2M0 Stage IIIA (according to the Japanese Classification of Gastric Cancer, 4th edition). Based on the pathology results, the patient was treated with postoperative adjuvant therapy with oral S-1. The patient maintained recurrence-free survival for 5.5 years postoperatively. However, 6 years postoperatively, the patient visited our department with cachexia. Computed tomography (CT) revealed a large amount of ascites and pleural effusion. He rapidly developed a poor circulatory and respiratory status and died 16 days after admission. An autopsy revealed severe bloody ascites and pleural effusion, as well as numerous nodules on the pleura and mesentery. Immunohistochemistry of the nodules revealed positivity for chromogranin A, Synaptophysin A, neural cell adhesion molecule (NCAM or CD56), and insulinoma-associated protein 1 (INSM1). The specimen showed a mixture of adenocarcinoma and neuroendocrine cell carcinoma and was diagnosed as MiNEN. Retrospective immunostaining of the surgical specimen showed similar results, and we diagnosed the patient with recurrence of EGJ-MiNEN.

**Conclusion:**

MiNEN has a poor prognosis; however, in some cases, long-term recurrence-free survival is achieved with radical resection and adjuvant chemotherapy.

## Background

Gastrointestinal neuroendocrine carcinoma (NEC) is a rare disease with a poor prognosis. The 4th edition of the WHO classification defines mixed adenoneuroendocrine carcinoma (MANEC) as tumors with > 30% each of regular adenocarcinoma and neuroendocrine carcinoma. This classification was revised in 2019, and the 5th edition of the WHO classification defines these tumors as mixed neuroendocrine-non-neuroendocrine neoplasms (MiNENs). Among gastrointestinal MiNENs, case reports of esophagogastric MiNEN (EGJ-MiNEN) are limited because of the rarity of esophagogastric MiNEN (EGJ-MiNEN).

For the treatment of resectable pancreatic and gastrointestinal NEC, the National Comprehensive Cancer Network (NCCN) guidelines recommend adjuvant chemotherapy and radiation therapy after radical resection or radical resection after preoperative chemotherapy and radiation therapy. Similarly, the European Neuroendocrine Tumor Society (ENETS) guidelines recommend surgery followed by adjuvant chemotherapy. However, evidence-based treatments for esophageal MiNENs have not yet been established. Continuous accumulation of cases is required to clarify these issues. Here, we present a case of drastic rapid recurrence and a fatal course 6 years after surgery.

## Case presentation

**Fig. 1:**
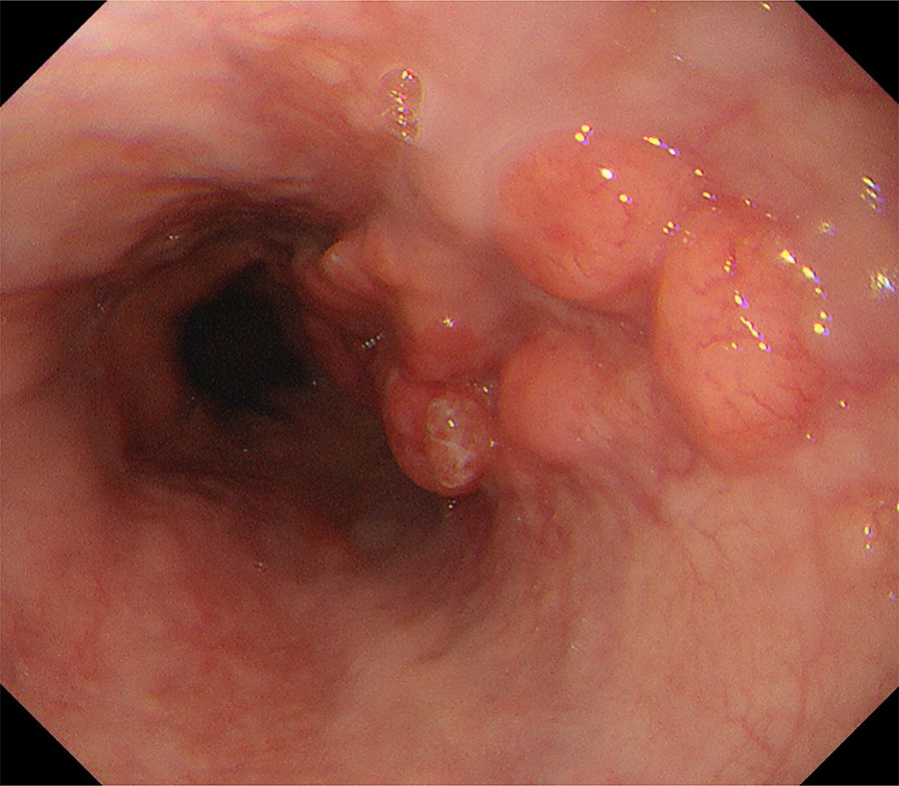
Esophagogastroduodenoscopy showing a 35 mm type 2 tumor at the esophagogastric junction. We classified this lesion as Siewert type I

**Fig. 2:**
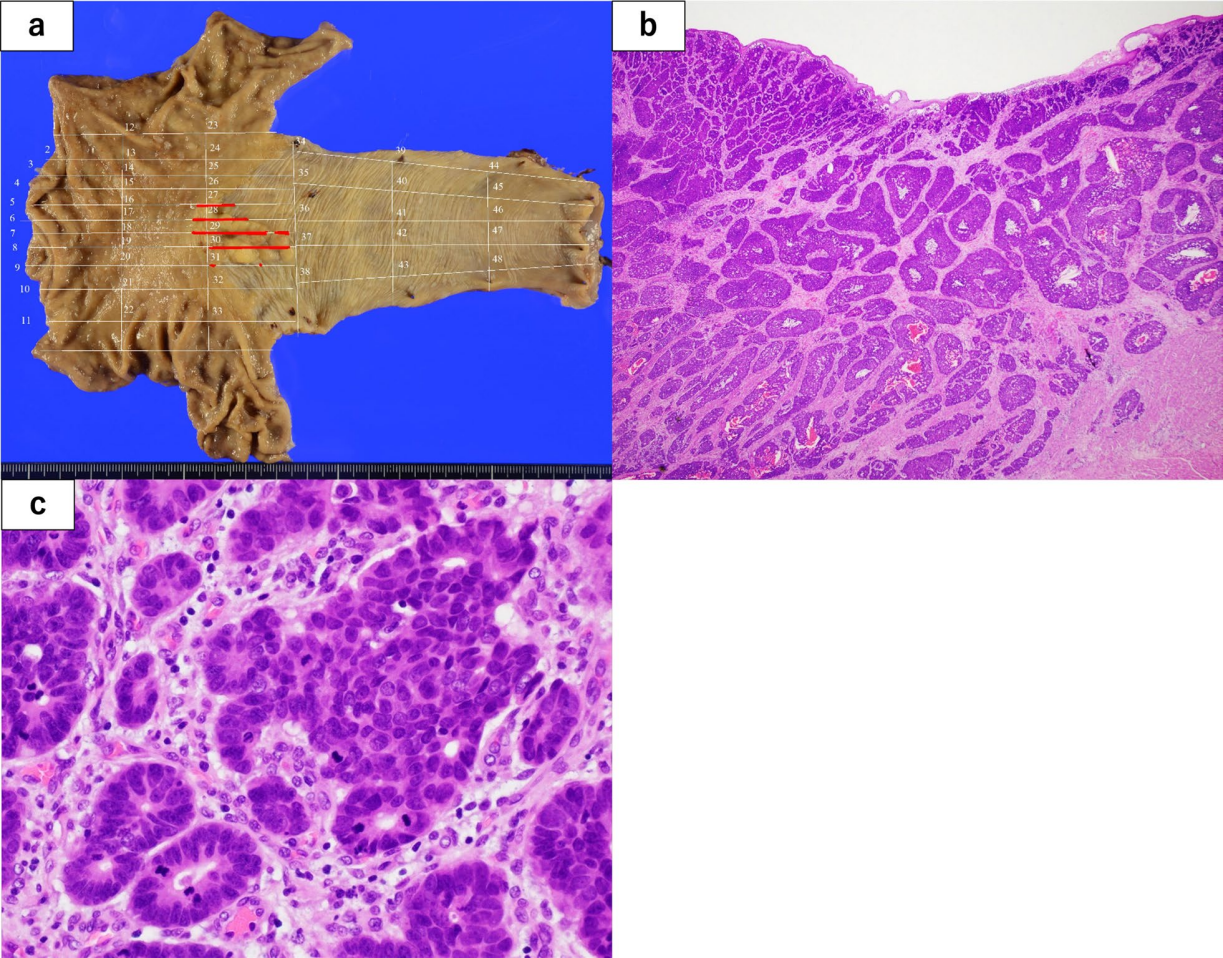
The tumor was a 35 mm type 2 lesion at the esophagogastric junction. The Siewert classification of this tumor was type I (**a**). The tumor partially invaded the subserosa and was diagnosed as pT3. No findings suspicious of a neuroendocrine neoplasm could be identified in the hematoxylin and eosin-stained postoperative specimen (**b** 1.25 × , **c** 20 ×)

A 58-year-old male presented to our department with epigastric pain. We performed several examinations, and esophagogastroduodenoscopy (EGD) revealed a type 2 tumor (Fig. 1) located on the proximal side of the EGJ, which was suspected to invade the muscularis propria. The biopsy result showed tubular adenocarcinoma. Computed tomography (CT) showed no regional lymph nodes or distant metastases. We performed thoracoscopic esophagectomy and gastric tube reconstruction via the posterior mediastinal route with two-field lymphadenectomy. The postoperative course was uneventful, and the patient was discharged on postoperative day 23. The pathological diagnosis was esophagogastric adenocarcinoma (35 × 20 mm, INFb, ly2, v3, pIM0, pT3 (A), pN2 (5/33), pPM0, pDM0, pRM0) (evaluated according to the Japanese Classification of Gastric Cancer, 4th edition) (Fig. 2a–c). Metastatic lymph nodes were located in the posterior mediastinal and lesser curvilinear regions. We did not perform additional immunostaining because there were no specific symptoms or findings of neuroendocrine neoplasm (NEN) at the time of diagnosis. We determined that the patient was at a high risk of recurrence and planned 1 year of postoperative adjuvant chemotherapy with oral S-1. We chose the adjuvant chemotherapy regimen based on the 4th edition of the Japanese Gastric Cancer Treatment Guidelines. Based on the diagnosis, we considered S-1 plus docetaxel therapy but selected S-1 therapy because of the hematologic toxicity and tolerability of the combination. Although we initially planned to administer adjuvant chemotherapy for 1 year, we discontinued it after 6 months because of adverse events. We continued outpatient follow-up, which included CT, EGD, and review of tumor marker levels. Until 5.5 years of postoperative follow-up, there were no findings suggestive of recurrence on any of the examinations, and the patient remained stable. However, 6 years postoperatively, the patient presented to our department with cachexia. CT revealed massive pleural effusion and ascites, but could not identify any other findings suggestive of recurrence. The patient was admitted to the hospital as an emergency, and multidisciplinary treatment was initiated. After admission, the patient complained of respiratory distress. We performed chest cavity drainage to improve the respiratory status, but did not achieve adequate improvement. No tumor cells were detected in the pleural fluid. Ten days after admission, the patient experienced cardiopulmonary arrest. Cardiopulmonary resuscitation was performed rapidly, and the patient had a return of spontaneous circulation and breathing. After resuscitation, the patient was admitted to the intensive care unit for catecholamine administration and placed on a ventilator. We continued these treatments but did not achieve sufficient improvement, and the patient died 16 days after admission.

**Fig. 3:**
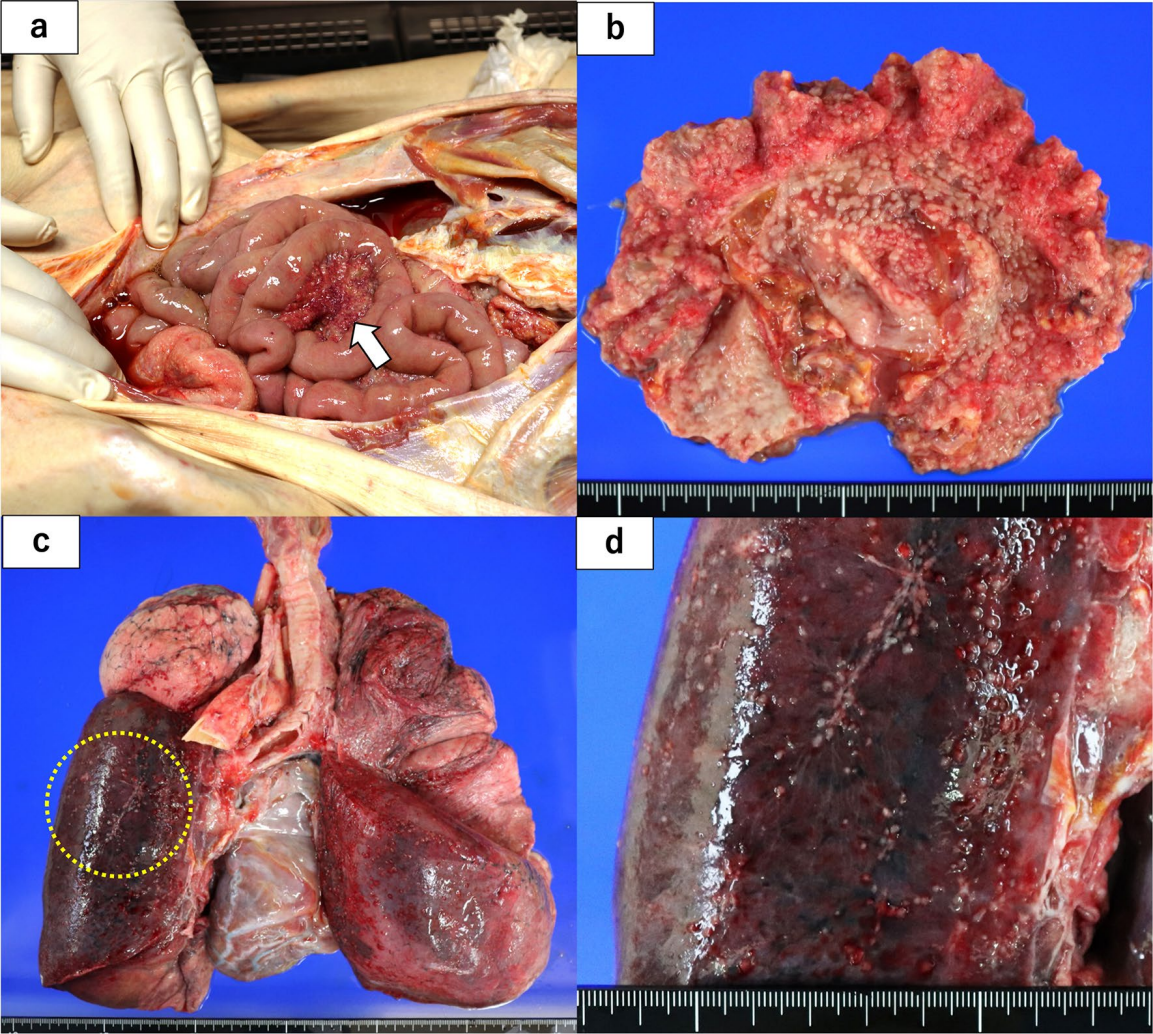
The abdominal cavity was filled with 700 mL of ascites. The abdominal wall and mesentery showed cancerous peritonitis with many disseminated nodules. No findings around the anastomosis were suggestive of recurrence (**a**). A specimen cut from the mesentery of the small intestine in (**a)** is shown in (**b)**. The microscopic findings of this lesion are described in (**b**). Pleural effusions were also observed bilaterally in the thoracic cavity. Micro-nodules were also spread on the lung surfaces (**c**). The image of (**d)** shows the circled area in (**c)**. Tumor cells similar to peritoneal dissemination were detected in these nodules, a finding consistent with carcinomatous pleurisy (**d**)

**Fig. 4:**
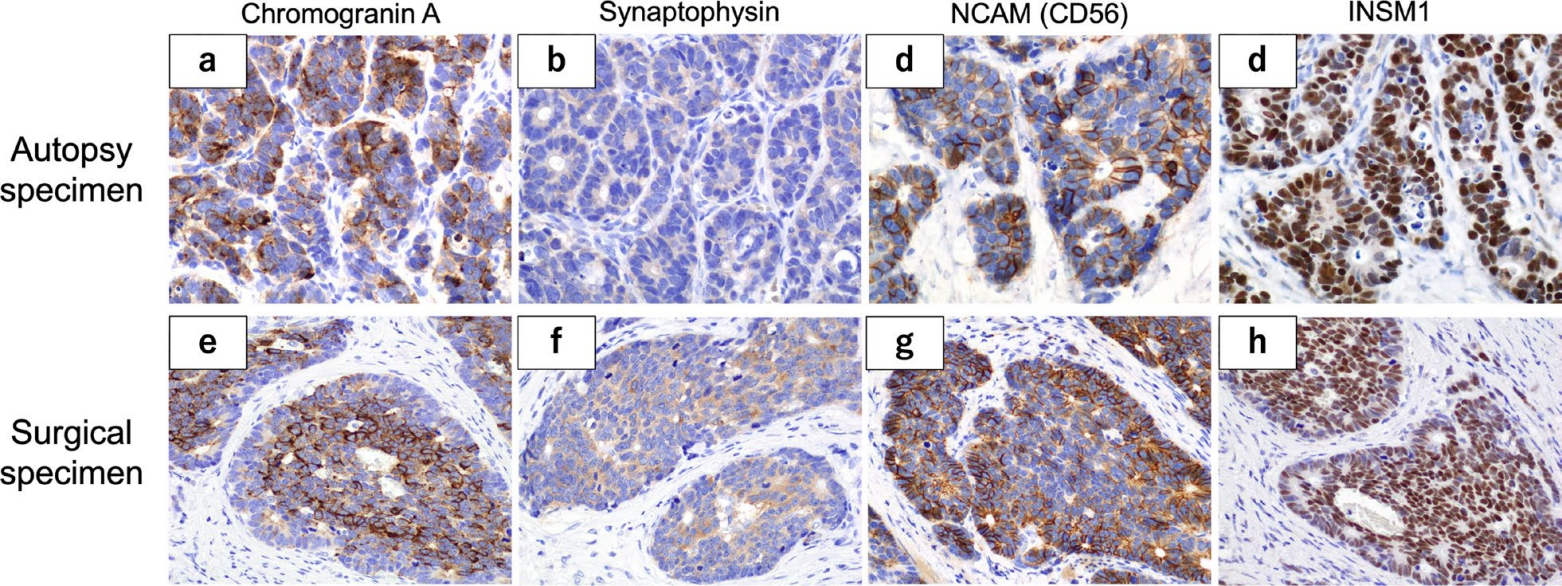
The immunostaining results of the specimens obtained during autopsy are shown in (**a–d**). Immunostaining of the peritoneal nodular lesions was positive for chromogranin A, synaptophysin A, neural cell adhesion molecule (CD56), and insulinoma-associated protein 1. These findings indicated that the lesion contained mixed neuroendocrine tumor cells. Histopathological findings showed more than 30% adenocarcinoma cells and 30% neuroendocrine neoplasm cells. Based on these findings, we diagnosed the patient with a mixed neuroendocrine–non-neuroendocrine neoplasm (**a–d**). We retrospectively performed additional immunostaining on the surgical specimen. The surgical specimen was positive for each neuroendocrine marker on immunostaining, similar to the autopsy specimen (**e–h**)

**Fig. 5:**
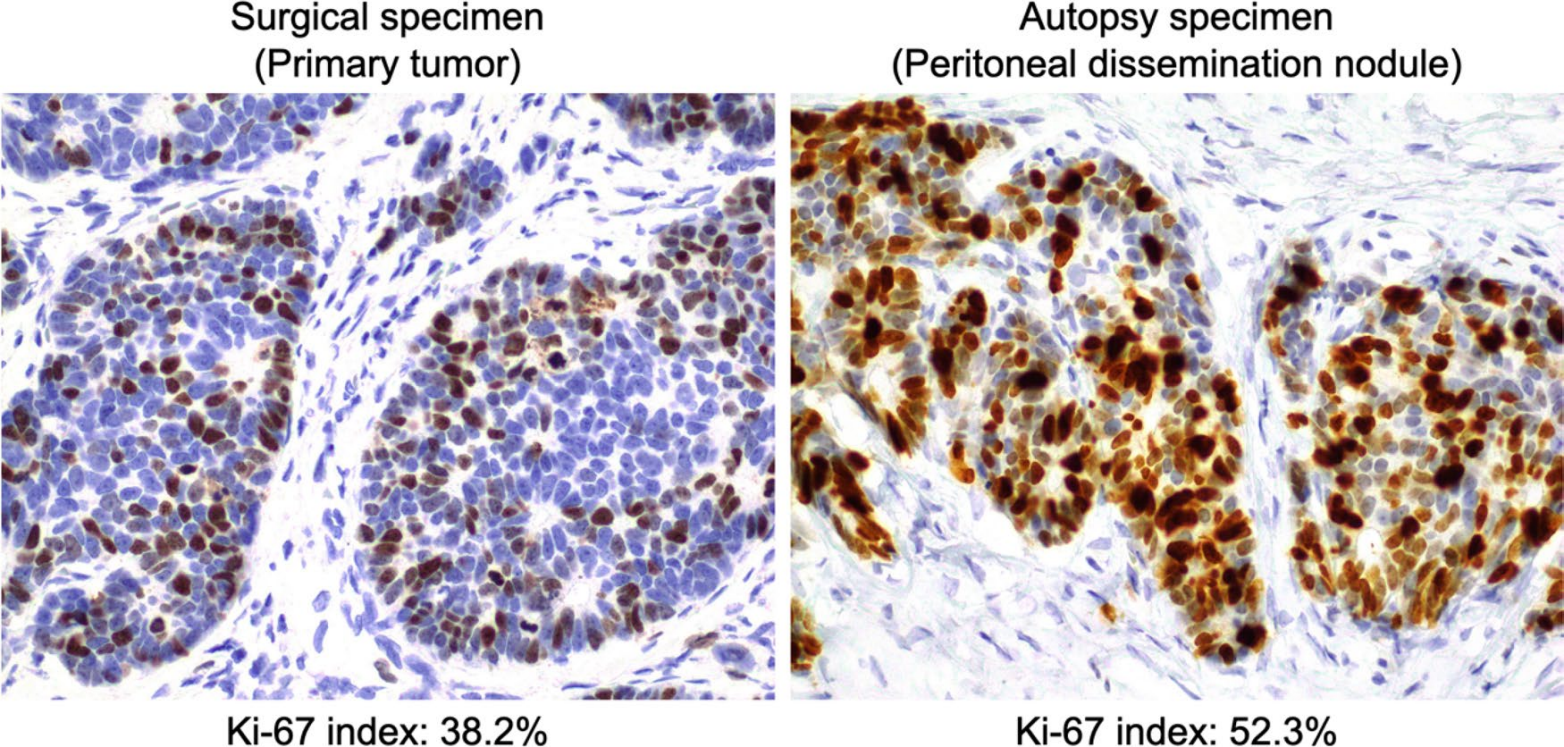
The Ki-67 index of the surgical specimens was 38.2%. The Ki-67 index of the pathological specimen was higher than that of the surgical specimen (52.3%)

**Fig. 6:**
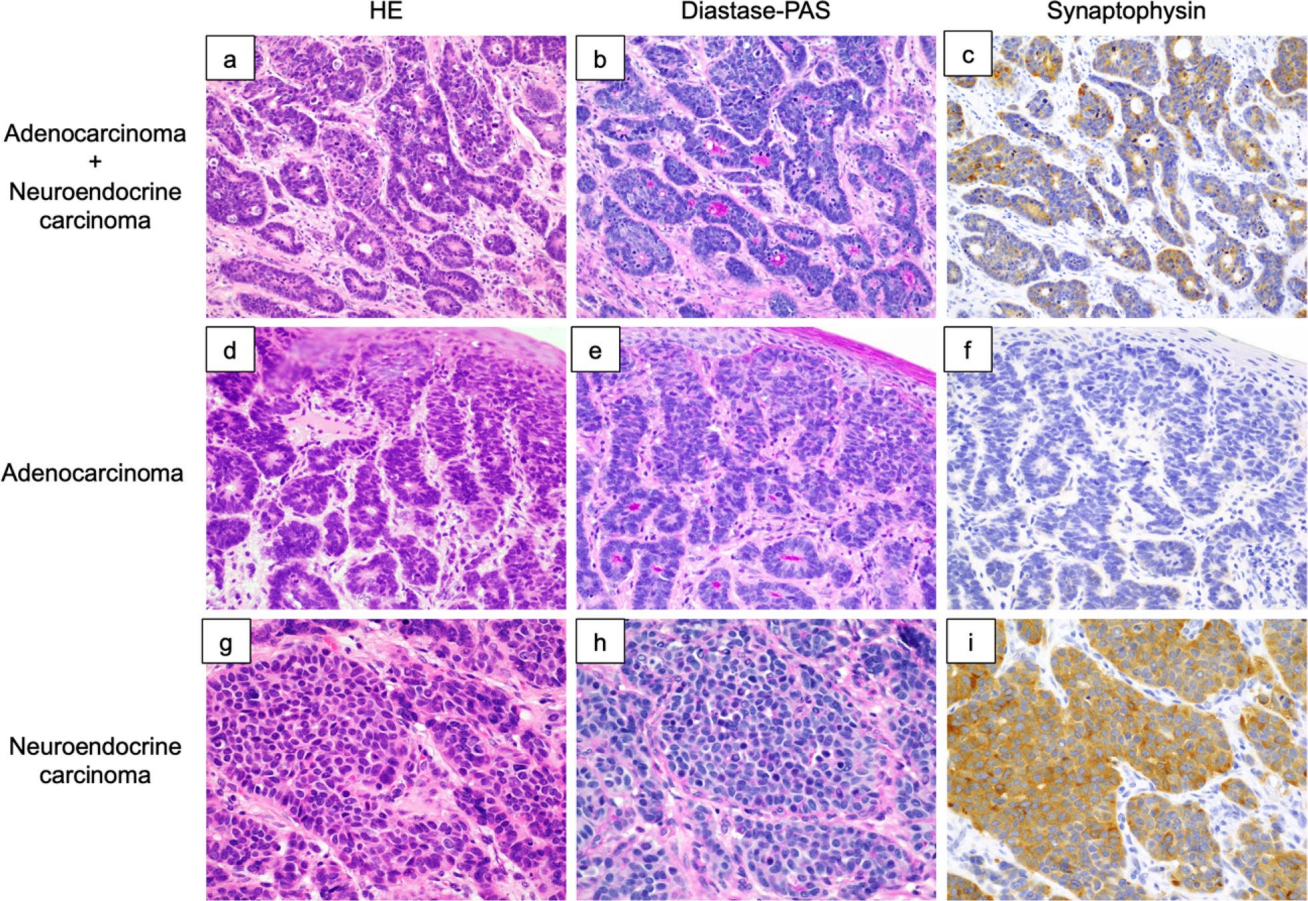
The majority of the regions in this tumor were synaptophysin (+), coinciding with areas forming diastase-PAS-stained (+) glandular duct structures (**a–c**). In contrast, normal adenocarcinoma areas (**d–f)** and NEC areas (**g–i**) were also present

To determine the cause of death, we performed an autopsy. We found a pale-yellow pleural effusion of 1000 ml in the right pleural cavity and 100 ml in the left pleural cavity. The abdomen contained 700 ml of pale bloody ascites. Adenocarcinoma cells were detected in both the ascites and pleural effusion. Observation of the abdominal cavity revealed numerous peritoneal seeding nodules in the mesentery of the small intestine (Fig. 3a, b). We also found micronodules on the surface of the lungs where adenocarcinoma cells were detected (Fig. 3c, d). There was no evidence of recurrence around the intestinal tract anastomosis. Histological evaluation of the peritoneal seeding nodules was consistent with adenocarcinoma on HE staining. However, the duration from surgery to recurrence and the rapid course after recurrence in the present case were atypical. Therefore, we performed several additional immunostaining procedures. These included chromogranin A (+), synaptophysin A (+), neural cell adhesion molecule (NCAM or CD56) (+), and insulinoma-associated protein 1 (INSM1) (+) (Fig. 4a–d). Surgical specimens were also immunostained with similar results (Fig. 4e–h). The Ki-67 index was 38.2%, while the pathologic autopsy specimen showed an increase to 52.3% (Fig. 5). Based on these results, we determined that the cause of death was cancer due to the recurrence of EGJ-MiNEN. An additional retrospective evaluation was performed on the surgical specimens. The majority of the regions in the surgical specimens were synaptophysin (+), coinciding with areas forming diastase-PAS stain (+) glandular duct structures (Fig. 6a–c). The tumor exhibited a complex composition, comprising three distinct areas: (1) areas displaying features of adenocarcinoma and NEC, (2) adenocarcinoma regions (Fig. 6d–f), and (3) NEC regions (Fig. 6g–i). Area (1) constituted over 70% of the tumor. The tumor displayed a mixture of amphicrine and collision tumor components, although a definitive classification was difficult.

## Discussion

MiNEN is a rare tumor with a poor prognosis. To the best of our knowledge, the present case achieved the longest recurrence-free survival rate among the reported cases (Table 1).

**Table 1:** Case reports of esophagogastric MANEC and MiNEN

	Author/year	Sex/age	Complain or symptom	Diagnosis	Siewert type	Method of treatment	Prognosis
1	Vetis [2] /2013	M/68	N.D	MANEC	N.D	ESD	N.D
2	Juanmartiñena [3] /2016	M/57	Dysphagia	MANEC	N.D	EG	N.D
3	Ambesh [4] /2017	M/67	Dysphagia	MANEC	II	EG	N.D
4	Yamamoto [5] /2018	M/81	Dysphagia	MANEC	I	Thoracoscopic-EG	Death (9 months from the surgery
5	Golombek [6] /2019	M/60	Upper abdominal pain	MANEC	I	Chemotherapy	Death (9 months from the diagnosis)
6	Riccò [7] /2020	M/66	N.D	MANEC	N.D	NAC (CBDCA + ETP) Lap-TG PAC(mFOLFOX)	Survival (4 years from the surgery)
7	Sulaiman [8] /2022	M/50	Dysphagia	MiNEN	N.D	NAC(CBDCA + PTX) EG	N.D
8	Lee [9] /2022	M/74	Anemia	MiNEN	I	BSC	N.D
9	Our case	M/58	Pericardial pain	MiNEN	I	Thoracoscopic-EG PAC(TS-1)	Death (6 years from the surgery

*N.D.* none to date, *ESD* endoscopic submucosal dissection, *NAC* neoadjuvant chemotherapy, *PAC* postoperative adjuvant chemotherapy, *EG* esophagectomy, *CBDCA* carboplatin, *ETP* etoposide, *Lap-TG* laparoscopic-assisted total gastrectomy, *mFOLFOX* modified oxaliplatin; 5-fluorouracil, and leucovorin therapy, *PTX* paclitaxel, *BSC* best supportive care

Neuroendocrine tumors were first described as carcinoids by Oberndorfer in 1907 [1]. At that time, the concept of carcinoid tumors ranged from benign to malignant, and as clinical studies progressed, the diversity of malignancy grades became apparent. The World Health Organization (WHO) reclassified carcinoids as neuroendocrine tumors (NET) in their 2000 classification. In their 2010 revision, NETs were reclassified as NETs (G1 and G2), NECs, and MANECs, based on Ki-67 expression and fission number. In their 2019 revision, MANEC was changed to the current MiNEN, which includes tumors other than adenocarcinomas.

We searched for the keywords “Esophagogastric MANEC” or “Esophagogastric MiNEN” in PubMed and found six case reports. MANEC and MiNEN are relatively new concepts; therefore, we did not include reports until these disease concepts were established. We summarized the data for these six cases and our case. All reported patients were male, with a median age of 60 years. Of these 7 patients, 5 underwent surgical treatment. Patients who underwent laparoscopic total gastrectomy received both neoadjuvant and postoperative adjuvant chemotherapy. This patient developed recurrence 7 months after surgery but achieved long-term survival with radiotherapy and the use of PD-1 inhibitors. Case 3 had liver metastasis; therefore, the patient underwent chemotherapy. The authors treated patients with multiple regimens according to the guidelines. Unfortunately, the patient died 9 months after the initial diagnosis.

For gastrointestinal NEC, localized disease is usually treated with radical resection. Radiotherapy may also be considered if the lesion is unresectable based on the patient’s background and tumor location. However, the high recurrence rate suggests that local therapy alone is inadequate. Casas et al. reported that patients treated with local therapy alone had a survival of 5 months, whereas those treated with local therapy plus systemic chemotherapy survived for 20 months in the small cell carcinoma of the esophagus group, indicating that the treatment strategy is an independent prognostic factor [10]. ENETS and NCCN provide guidelines for NEC treatment. ENETS recommends surgery plus postoperative adjuvant chemotherapy with platinum-based drugs as a treatment option for NEC [11]. The NCCN recommends either resection plus adjuvant chemotherapy with or without radiation therapy or preoperative chemotherapy with or without radiation therapy plus surgery [12]. There is no evidence of a treatment strategy specific to EGJ-MiNEN; however, adjuvant therapy should be added after radical resection, as in other NENs. There are limited case reports and insufficient data to establish clear evidence for the benefits of neoadjuvant chemotherapy (NAC). Esophagectomy is highly invasive; therefore, the initiation of postoperative adjuvant therapy is likely to be delayed or impossible. NAC is known to be effective in treating esophageal cancer. Similarly, the effectiveness of NAC in EGJ-MiNENs requires further investigation.

Based on these guidelines, local therapy for EGJ-MiNENs should be administered with preoperative or postoperative chemotherapy in mind. Common regimens for gastrointestinal NEN include cisplatin plus etoposide (EP) and cisplatin plus irinotecan (IP) [11, 12]. EP has been reported to have a response rate of approximately 30% when used as first-line therapy for advanced gastrointestinal NEC/MiNEN [13]. JCOG 1213 reported that EP and IP regimens were effective and that there was no difference in response rate in gastrointestinal NEC [14]. Similarly, a retrospective study of IP treatment for gastrointestinal and pancreatic NEC at 23 centers in Japan showed that it was not inferior to EP [15]. Therefore, postoperative adjuvant therapy with these regimens should be considered for EGJ-MiNENs.

In this case, NEN was not actively suspected immediately after surgery, and the patient was diagnosed with MiNEN via pathological autopsy. Therefore, based on the patient’s physical status and adenocarcinoma histology, we treated the patient with adjuvant S-1 oral chemotherapy according to the postoperative adjuvant chemotherapy in the Japanese guidelines for gastric cancer. The planned treatment duration was initially 1 year; however, adjuvant therapy was discontinued after 6 months because of adverse gastrointestinal events. If the surgical specimen had been diagnosed with MiNEN, postoperative adjuvant therapy would have been considered based on ENETS and NCCN guidelines. However, in this case, the primary tissue of the metastatic lymph nodes was adenocarcinoma. Therefore, we believe that even with a diagnosis of MiNEN, we would have chosen the S-1 regimen. If the lymph node metastatic tissue contained NEC, regimens such as EP or IP would have been selected. There are no reports of adjuvant chemotherapy with oral S-1 for EGJ-MiNEN; therefore, there is no clear evidence for treatment. Yamaguchi et al. treated 11 patients with advanced gastrointestinal NEC/MiNENs in whom EP or IP was ineffective with oral S-1 as second-line therapy, and the OS was 12.2 months, which is a promising therapeutic effect to a certain extent [15]. Gastrointestinal NEC has a poor prognosis, with a survival of 38 months for localized disease and 5 months for metastases [16]. However, this case led to a long-term recurrence-free survival of more than 5 years. This long-term survival may have been related to postoperative adjuvant therapy with oral S-1, in addition to the achievement of complete resection. EP and IP are not always feasible for all patients because of hematological and gastrointestinal toxicities and renal dysfunction. In such cases, there is a possibility that S-1 may be an additional option for postoperative adjuvant chemotherapy for EGJ-MiNEN.

## Conclusion

Esophageal MiNEN is a rare tumor that is difficult to diagnose without immunostaining. Therefore, esophageal MiNEN should be suspected at the time of the initial diagnosis. In some resectable cases, definitive radical resection and adjuvant therapy may improve long-term prognosis. More case reports are needed to improve the prognosis of esophageal MiNENs.

## Data Availability

All data generated during this study are included in this published article.

## Abbreviations

MiNEN: Mixed neuroendocrine–non-neuroendocrine neoplasm
EGJ-MiNEN: Esophagogastric junction MiNEN
NEC: Neuroendocrine carcinoma
MANEC: Adenoneuroendocrine carcinoma
NCCN: National Comprehensive Cancer Network
ENETS: European Neuroendocrine Tumor Society
EGD: Esophagogastroduodenoscopy
CT: Computed tomography
NEN: Neuroendocrine neoplasm
NCAM: Neural cell adhesion molecule
INSM1: Insulinoma-associated protein 1
NET: Neuroendocrine tumor
EP: Cisplatin plus etoposide
IP: Cisplatin plus irinotecan
